# A rare case of primary chordoma of the nasopharynx mimicking nasopharyngeal carcinoma: A case report

**DOI:** 10.1016/j.ijscr.2024.110779

**Published:** 2024-12-24

**Authors:** Teketel Tadesse Geremew, Ghion Getenet Engida, Adugnaw Kindu Mihiret, Abeje Diress Gelaw, Tigist Gutema Tesgera

**Affiliations:** a Department of Pathology, Hawassa University Comprehensive Specialized Hospital, Hawassa, Sidama, Ethiopia; b Department of Internal Medicine, University of Gondar, College of Medicine and Health Science, Ethiopia; c Department of Radiology, University of Gondar, College of Medicine and Health Science, Ethiopia; d Department of Internal medicine, Hawassa University Comprehensive Specialized Hospital, Hawassa, Sidama, Ethiopia

**Keywords:** Chordoma, Nasopharyngeal, Notochord, Nasopharyngeal carcinoma, Case report

## Abstract

**Introduction and importance:**

Chordoma is an uncommon malignant tumor that originates from the remnants of the primitive notochord in the embryo. They account for 1 % of intracranial tumors and 4 % of primary bone tumors. It is a locally aggressive tumor with a low risk of metastasis. It is more common in males than in females and is more common in the age range of 40–60. It most commonly occurs in the sacrococcygeal areas but can occur in uncommon locations, such as the dental area and nasopharynx. This case is important because there are few case reports in the world.

**Case presentation:**

Here we present a 60 years old male patient presented with foul smelling nasal discharge, nasal obstruction and snoring of 2 years duration and Head CT scan was concluded to be advanced nasopharyngeal carcinoma but histopathology result turned out to be the uncommon nasopharyngeal chordoma. Therefore, he was treated with surgical excision and adjuvant radiotherapy.

**Clinical discussion:**

Patient presentation usually varies by location; however, patients can be asymptomatic. CT and MRI are considered the initial modes of investigation, but histopathology is needed to confirm the diagnosis. Surgery is the primary mode of treatment with or without adjuvant chemoradiotherapy.

**Conclusion:**

Even though the typical location of chordoma is the sacrococcygeal area of the body, it can arise in uncommon locations, such as the nasopharynx.

## Highlights

• Chordoma is an uncommon malignant tumor that originates from the remnants of the primitive notochord in the embryo.
• They account for 1 % of intracranial tumors and 4% of primary bone tumors.
• It most commonly occurs in the sacrococcygeal areas but can occur in uncommon locations, such as the dental area and nasopharynx.
• It is a locally aggressive tumor with a low risk of metastasis.

## 1 Introduction

Chordomas are rare malignant tumors that originate from the remnants of the primitive notochord in the embryo, which is a primitive cell line around the skull base and vertebral column. Remnants of the notochord persist in or near the midline, which is closed by bone. Chordomas are categorized as locally invasive, but rarely metastasize [1]. It affects males commonly than female at a 2:1 ratio, with an age range of 40–60 years [2,3]. Chordomas represent 1 % of intracranial tumors and 4 % of all primary bone tumors. Intracranial chordomas account for 1/3 of all chordomas and occur in the vicinity of the clivus (sphenooccipital bone) [4]. Common sites for chordoma include the sacrococcygeal (50 %), craniocervical/spheno-occipital (35 %), and thoracolumbar spine (15 %). Craniocervical chordomas most often involve the dorsum sellae, the clivus, and the nasopharynx [2]. Unusual locations have been reported, including the mandible and maxilla, which are called dental chordomas; some also have nasal and paranasal presentations [5].

The clinical signs and symptoms differ depending on the anatomical location of the tumor [6]. Computed tomography (CT) and magnetic resonance imaging (MRI) are usually required to evaluate the chordomas [7]. Chordomas are considered to have a poor sensitivity to radiotherapy and chemotherapy; however, surgery is the treatment of choice [7,8].

This case report is written following the SCARE criteria [9].

## 2 Case

A 60-year-old male patient, who has presented with foul smelling discharge through the nose, headache, difficulty of breathing through the nose worsens during sleeping position associated with this he has snoring and mouth breath for 2 years. Currently, our patient complained of swelling of the left lower eyelid to the extent that he had difficulty opening his left eye. The patient had no history of trauma or swelling to other site. He had no history of diabetes mellitus, hypertension, or an immunocompromised status. He looks well nourished.

### 2.1 Physical findings

On general appearance, the patient looked chronically sick.

Vital signs were all within the normal limits.

**Fig. 1:**
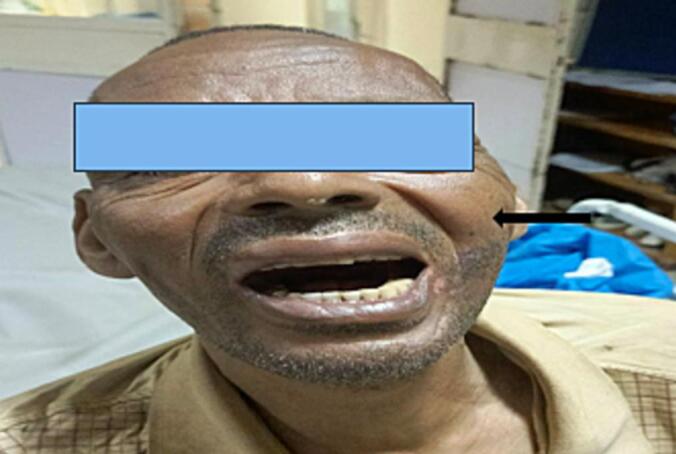
Non tender asymmetrical swelling over left face(black arrow).

HEENT examination revealed a 5 × 4 cm non-tender, diffuse swelling over the left lower eyelid that pushed the eyelid (Fig. 1).

Lymphoglandular system: No significant lymphadenopathy.

### 2.2 On investigation

Full blood count (WBC-7.6 × 10^9^/L, Hgb-11.1 g/dl, and platelet count 275 × 10^9^/L). Liver function test, renal function test and total protein were within the normal range.

**Fig. 2:**
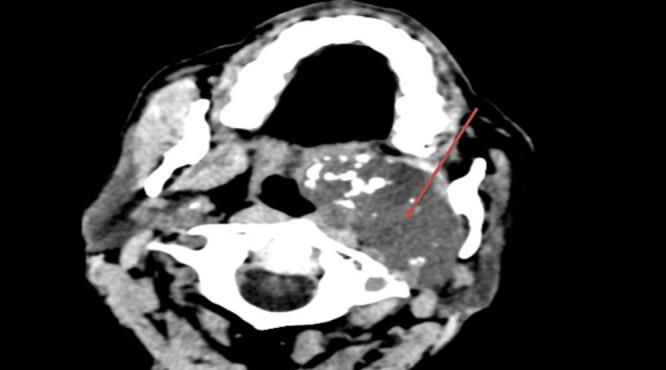
7 × 6.6 × 5 cm mass with mild and heterogenous post contrast enhancement.

**Fig. 3:**
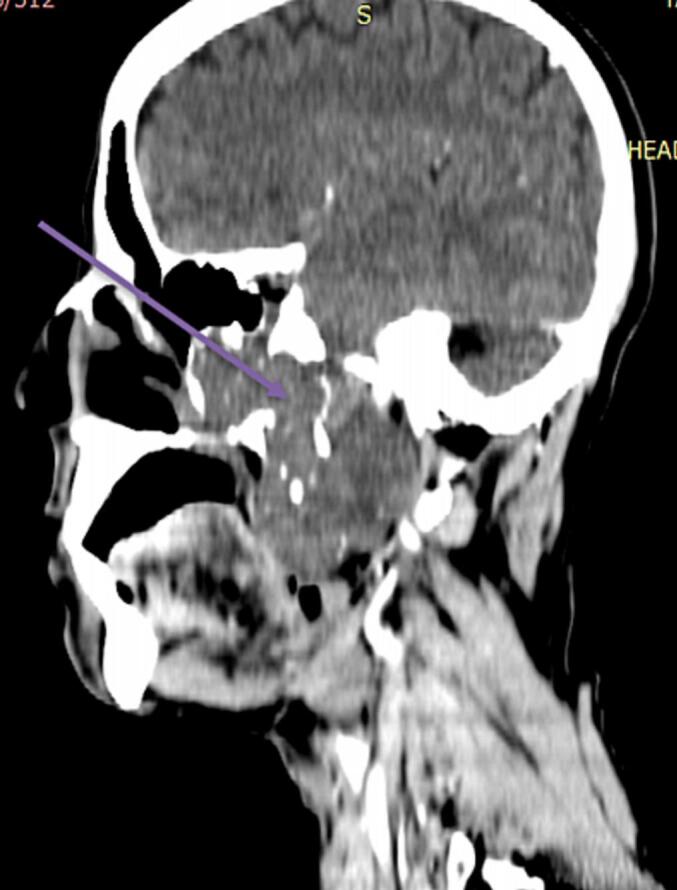
7 × 6.6 × 5 cm mass with mild and heterogenous post contrast enhancement extend in to left nasal cavity, inferiorly into upper part of oropharynx pushing soft palate with extension into maxillary sinus.

Left Head & PNS CT revealed a: 7 × 6.6 × 5 cm mass with mild and heterogeneous post-contrast enhancement (Fig. 2, Fig. 3). On the basis of this CT impression, the patient was diagnosed with advanced Nasopharengeal carcinoma.

Following the above provisional diagnosis, the patient and his family were counselled for wide surgical excision. Hence, under spinal anesthesia, an excisional biopsy with a possible gross tumor-free margin was performed, and the specimen was sent for histopathological confirmation.

**Fig. 4:**
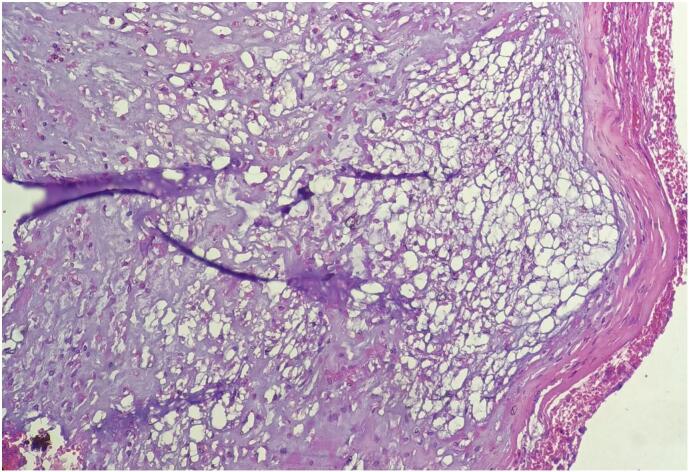
Low power view shows (4×) lobules, solid sheets physaliphorous cells with bubbly cytoplasm.

**Fig. 5:**
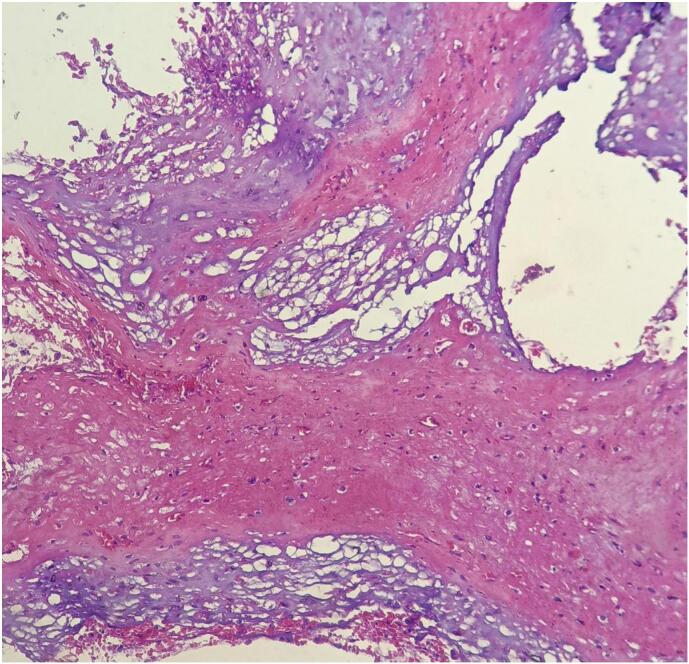
Medium power view (20×) shows lobules, solid sheets of physaliphorous cells with abundant bubbly cytoplasm and forming cord like architecture.

**Fig. 6:**
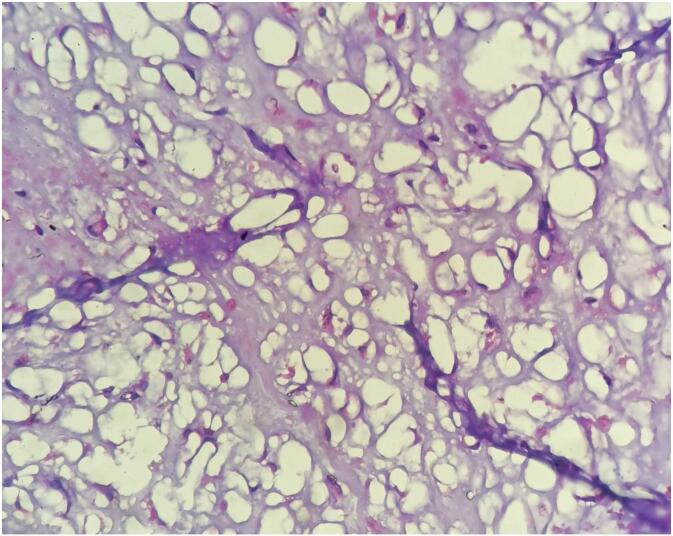
High power view (40×) shows lobules, solid sheets of physaliphorous cells with polygonal and hyperchromatic nucleus abundant bubbly cytoplasm and forming cord like architecture.

Microscopic examination of the lesion revealed lobulated tissue composed of solid sheets and cords of polygonal cells with abundant bubbly cytoplasms. The patient was diagnosed with conventional nasopharyngeal chordoma (Fig. 4, Fig. 5, Fig. 6). IHC which is s100 was done and it was positive.

Subsequently, he was put on proton radiation therapy, and he has been on follow-up, came to the follow-up clinic twice during the subsequent three months and with no significant lesions or complaints.

## 3 Discussion

Chordoma is an uncommon neoplasm that arises from the remnant of the primitive notochord in the embryo. It is locally aggressive and commonly develops along the craniospinal axis [4]. It was first described by Virchow in 1857 as a tumor composed of vacuolated, physaliphorous cells derived from the rest of the embryonic notochord, along the midline central nervous system axis [10]. The notochord is a rod-like aggregate of cells extending over the entire length of the embryo on the midline ventral to the developing neural tube. The embryonic notochord degenerates early in fetal development after being surrounded by sclerotome mesenchymal cells and remains in the nucleus pulposus within the inter-vertebral disc. Occasionally, residual notochord cells remain outside of the inter-vertebral disc and may become neoplastic [11]. The prevalence of chordoma is <0.1 per 100,000 individuals per year and it constitutes 1–4 % of all primary bone tumors [4] Chordomas can manifest in individuals of any age group. However, most cases have been identified in adults and rarely in children [6].

The most common location of chordoma is the sacrum, which accounts for 50–60 % of the cases, followed by the skull base, which accounts for 25–35 % commonly around the sphene-occipital area. The cervical and thoracolumbar vertebrae account for 10 % and 5 % of the cases, respectively [12].

The clinical signs and symptoms differ depending on the anatomical location of the tumor. Symptoms may vary with vital structure compression, headaches, visual field defects, or obstruction if they extend into the nasopharyngeal area [6], as in our case. Occasionally, the onset is asymptomatic; therefore, the early diagnosis of chordoma is difficult. Computed tomography (CT) and magnetic resonance imaging (MRI) are usually required to evaluate the chordomas. Owing to the involvement of the bone, a CT scan is needed, and for the involvement of nearby structures, MRI imaging is needed. MRI is the gold standard modality used in the evaluation of pre- and post-treatment is MRI imaging. On a CT scan, the tumor appears centrally located with expansile soft tissue that originates from the clivus, with destruction of the lytic bone. On T2-weighted MRI, a high signal intensity was observed [4,13].

Differential diagnosis differs according to the location of the mass. In this case, the differential diagnosis was different from that of classical chordoma [14]. Therefore, the differential diagnosis in our case was vascular lesions such as hemangiomas, cysts, and nasopharyngeal malignancies. Chordomas can be classified into three categories: classical (conventional), chondroid, and dedifferentiated. The classical (conventional) type is slow growing and consists of physaliphorous cells within a myxoid matrix that may show cellular proliferation. Furthermore, the chondroid type resembles hyaline cartilage neoplasm. Moreover, the dedifferentiated type is rapidly growing and can lead to metastatic spread with the worst prognosis and consists of a mesenchymal component and a sarcomatoid appearance. Immunohistochemistry revealed that the chordoma was positive for the S100 protein, vimentin, epithelial membrane antigen, and cytokeratins [6,14,15].

Although CT and MRI features are nonspecific, they may be suggestive of chordomas, including a midline location and expansible or lobular soft tissue masses with well-defined margins. Other nasopharyngeal malignancies may destroy the clival bone but do not demonstrate this midline tract. CT is ideal for evaluating bony involvement, whereas MRI is useful for evaluating the surrounding soft tissues and their extension into adjacent structures [16]. Chordomas are considered to have a poor sensitivity to radiotherapy and chemotherapy; however, surgery is the treatment of choice [8,10].

Surgery is the primary modality for the management of chordomas. Local recurrence rates, in addition to survival rates, are dependent on the achievement of negative surgical margins, with recurrence rates on the order of 70 % in cases where negative margins are not achieved [2,12]. In a series of 52 patients, Boriani et al. reported that 100 % of patients treated with radiation alone, palliative therapy, or intralesional intracapsular excision had local recurrence within 17–20 months. However, only 20 % of patients treated with en bloc resection with appropriate margins had local recurrence at 56–94 months [17]. The usefulness of radiotherapy as primary or adjuvant treatment has been debated. In contrast, some investigators have reported a slight effect of RT. In contrast, other studies have reported improved local control and prolonged disease-free survival after radiotherapy as an adjuvant treatment [18]. This patient underwent surgical excision with possible free surgical margins and adjuvant chemotherapy was administered.

## 4 Conclusion

Even though the typical location of chordoma is the sacrococcygeal area of the body, it can arise in uncommon locations, such as the nasopharynx. Therefore, Doctors and radiologists should have a high index of suspension of chordomas in locally aggressive masses. Differentiating nasopharyngeal carcinoma with nasopharyngeal chordoma may be difficult on imaging and clinical presentation alone; therefore, histopathologic correlation with subsequent IHC is very helpful because of the prognosis as well as the treatment for the two conditions.

## Author contribution

Teketel Tadesse Geremew, MD - Study concept and design, writing the paper, literature review and editing and critical review of the paper

Ghion Getenet Engida, Adugnaw Kindu Mihiret and Abeje Diress Gelaw, MD - Involved in acquisition of data, literature review of the paper, writing and drafting the paper, editing and critical review of the paper

Tigist Gutema Tesgera MD - literature review of the paper, writing and drafting the paper, editing and critical review of the paper

## Consent

Written informed consent was obtained from the patient, by their native language, for publication of non-identifying information including accompanying intraoperative images. A copy of the written consent is available for review by the Editor-in-Chief of this journal on request.

## Ethical approval

Ethical approval is deemed unnecessary by the Hawassa university collage of medical and health science ethical committee as this is a single case encountered during practice and it doesn't involve human or animal experiment.

## Guarantor

Teketel Tadesse Geremew, MD.

## Research registration number

Not applicable.

## Funding

None.

## Conflict of interest statement

The authors declare that there are no conflicts of interest on this case report.
